# Contribution of next generation sequencing to the diagnosis of inborn errors of immunity in a pediatric cohort

**DOI:** 10.3389/fimmu.2025.1638544

**Published:** 2025-10-20

**Authors:** Guadalupe Fernanda Godinez-Zamora, Patricia Baeza-Capetillo, Omar Josué Saucedo-Ramírez, Blanca Estela Del-Río-Navarro, Sara Elva Espinosa-Padilla, Verónica Fabiola Morán-Barroso, Jesus Aguirre-Hernandez

**Affiliations:** ^1^ Laboratorio de Genómica, Genética y Bioinformática, Hospital Infantil de México Federico Gómez, Mexico City, Mexico; ^2^ Programa de Maestría y Doctorado en Ciencias Médicas Odontológicas y de la Salud, Universidad Nacional Autónoma de México (UNAM), Mexico City, Mexico; ^3^ Servicio de Alergia e Inmunología Clínica, Hospital Infantil de México Federico Gómez, Mexico City, Mexico; ^4^ Unidad de Investigación en Inmunodeficiencias, Instituto Nacional de Pediatría, Mexico City, Mexico; ^5^ Departamento de Genética, Hospital General de México Dr. Eduardo Liceaga, Mexico City, Mexico

**Keywords:** inborn errors of immunity (IEI), whole exome sequencing (WES), molecular diagnosis, novel genes, international union of immunological societies (IUIS), diagnostic yield, children

## Abstract

Inborn errors of immunity (IEI) number more than 500 diseases, with most affected patients being children. Their precise diagnosis is hampered by overlapping phenotypes, and by their ample and varied phenotypic spectrum. We analyzed the contribution of next generation sequencing to the diagnosis of IEI in a cohort of 157 children in a referral hospital in Mexico City. Following the classification of the International Union of Immunological Societies (IUIS), patients were assigned to an IEI group before sequencing, or to an “undefined” group, if it was not possible to assign them to any of them. Patients were again classified in the IUIS groups after sequencing. The diagnostic yield was 32.48%. Before sequencing, the largest group was comprised by patients that could not be assigned to a specific IUIS group (38.35% of the cohort), while after sequencing the largest group was made by the patients where no likely molecular diagnosis was found (67.52% of the cohort). Patients that were assigned to an IUIS group were confirmed to have a disease of that same group in 31.25% of the cases, while in 10.42% the molecular diagnosis corresponded to an immunodeficiency of a different group to the one initially suggested. In 18.03% of the children that could not be assigned to an immunodeficiency group before sequencing, a molecular diagnosis was reached after sequencing. In the patients that remained without a molecular diagnosis, the possibility of new IEI genes was explored by analyzing the variants, first in a curated set of immune related genes, and then across the whole exome. However, after filtering the variants, by frequency, predicted consequence, and known biology, no new IEI candidate genes were identified. This results underscore the large impact of next generation sequencing for the correct diagnosis of IEI, and also points to the need to better understand their genetic architecture in order to increase the diagnostic yield.

## Introduction

Inborn errors of immunity (IEI), formerly known as primary immunodeficiencies, are a group of monogenic diseases characterized by dysregulation of the immune system that may affect the innate and the adaptive systems, as well as multiple cellular functions (1). Symptoms may present themselves from a very early age, or during childhood (2). However, recent studies suggest that in the adult population these diseases may be more common than previously thought, and their incidence, across all age groups, may be as high as 1:5,000 (3, 4).

IEI have a broad phenotypic spectrum and variable expressivity (5). These diseases may stem from alterations in a large number of genes (6). Different alterations in the same gene may lead to different phenotypes, and sometimes to different diseases. On the other hand, alterations in different genes may result in different diseases that nevertheless show overlapping phenotypes, making it difficult to distinguish between them (7, 8). Due to these characteristics, next generation sequencing (NGS) has been increasingly used for diagnostic purposes as it enables the simultaneous interrogation of a large number of genes, with the concomitant savings in time and money this entails (5, 9, 10).

Since 2014, an increasing number of research groups have been studying cohorts of IEI patients using NGS, both for diagnostic purposes, and to identify new genes associated with these diseases. In some studies, a panel of candidate genes was sequenced, while in others the whole coding exome (WES), and even the whole genome (WGS), were studied. More than 35 cohorts, studied by NGS, have been reported in the literature (3, 11–19). Most of these studies have been done with a diagnostic goal in mind, while only a small number have included a search for new IEI genes. Up to 2018, most of these studies involved sequencing of gene panels (20–22), while later on WES became increasingly important (11, 12, 14). More recently, particularly in well-resourced institutions, WGS has also been deployed (3, 9, 23, 24). Most NGS studies of large cohorts have been undertaken in European countries and the USA, although other parts of the world are also represented (12, 16–18, 21, 22, 25–27). In these cohort studies, the diagnostic yield varied widely, from 15 to 79% (9, 13, 19). These differences in diagnostic yield are the result of both, the NGS approach used (e.g., gene panels, WES, or WGS), and the criteria employed for selecting the patients to be sequenced. In this regard, some cohorts comprised patients of a single IEI group, while in other studies a suspicion of an IEI was enough to include the patient in the cohort. In the former type of studies, the diagnostic yield is higher than in studies with more heterogeneous diseases. On average, the diagnostic yield is 30% (11). There are various reasons for this relatively low diagnostic yield. Among others, the fact that genes underlying these highly genetically heterogenous diseases still remain to be identified, as underscored by the regular addition of new genes to the literature (6, 9, 14, 28–35). The most recent update of the IUIS contains 555 conditions and 504 genes (6).

Some groups have studied large cohorts of patients with IEI diseases, both to arrive at the molecular diagnosis and to search for new candidate genes. Following this approach, Stray-Pedersen et al. (14) identified six new candidate genes after using WES to study a large number of sporadic cases. With WGS and a Bayesian approach in a large cohort of sporadic cases, Thaventhiran et al. (3) also identified several new candidate genes and pointed to the interplay between high-penetrance rare monogenic variants and common variants. Itan and Casanova (36) used a different approach to search for new genes associated with IEI; they studied the human connectome of IEI genes to propose new candidate genes. In subsequent years, some of these have indeed been found to underly new IEI.

In this paper, we present the first large cohort of IEI patients in Mexico, studied by NGS for diagnostic purposes, followed by the search for new candidate genes: first in a curated list of possible IEI genes, and then by studying variants across the whole exome.

## Materials and methods

We studied a cohort of 157 pediatric patients, from Hospital Infantil de México Federico Gómez, between June 2015 and May 2024. These patients were suspected of having an IEI whose identity could not be precisely determined by laboratory tests; in other cases, a molecular diagnosis was required before deciding on the appropriate treatment. The information available for each patient was variable. In some, it was mostly limited to the clinical history (e.g., repeated infections at a very early age); for some others, results of a few laboratory tests were available.

DNA was extracted from peripheral blood samples, or from buccal swabs. In nine patients the TruSightOne Targeted Regions panel v1.1 (Illumina) was used to obtain the sequencing library. From 2016 to 2023, WES was done in 125 patients with the Nextera Rapid Capture Exome Targeted Regions v1.2 kit (Illumina). The Twist exome probes, which were developed more recently, seem to provide better coverage of the targeted regions through better uniformity (37), so starting in 2023 we used Twist ILMN Exome 2.0 or 2.5 Plus Panel kits (Illumina) to sequence the exome of 23 patients. Paired end sequencing (150 bases per end) was done in a NextSeq 500 instrument (Illumina; RRID: SCR_014983). Read alignment and variant calling were undertaken with BaseSpace Enrichment Workflow (Illumina). Quality control was performed by filtering variants with “PASS” in the FILTER field, with a minimum depth of 10X, and a variant allele fraction of 0.20 or higher; this was done with bcftools (RRID: SCR_005227) (38) and the command: bcftools view -O z -f “PASS” -e ‘INFO/DP<10’ -e ‘(AD[0:1]/(AD[0:0]+AD[0:1]))<2/10’. Exomiser (RRID: SCR_002192) was used to annotate, filter, and prioritize genes and variants (39), with human phenotype ontology (HPO, RRID: SCR_006016) terms obtained from the information provided by the clinicians (40). To carry out the analysis, a list of IEI genes was provided, comprising all the genes in the most current update of the IUIS at the time of the analysis (4, 10, 41, 42). The list was supplemented with the genes in the Primary Immunodeficiency panel in PanelApp (43, 44), plus any new IEI genes added to OMIM (RRID: SCR_006437) (45). From this list, three virtual subpanels were created, one for each mode of inheritance (biallelic, monoallelic, and X-linked). For each patient, Exomiser was run separately with each one of these subpanels, using the default parameters set for each mode of inheritance. If variants and genotypes of interest were found, they were reannotated with Variant Effect Predictor (VeP, RRID: SCR_007931) (46), reads were visually examined with Integrative Genomics Viewer (IGV, RRID: SCR_011793) (47, 48), and variants were searched in an in-house database. The possibility of digenic causation was also considered, and Exomiser was run with a set of genes obtained from the DIDA database and the literature (49, 50).

If no molecular diagnosis was reached, the possibility of the patient not having an IEI was considered, and Exomiser was run with a larger set of genes made by merging the Pediatric Disorders, Severe Pediatric Disorders, and Pediatric Disorders Additional Genes panels of PanelApp. If still no likely diagnosis was identified, a further analysis was done with all variants in genes known to be associated with a disease.

In patients where a molecular diagnosis was unsuccessful, a search for new IEI candidate genes was attempted. For this purpose, a list of 3,938 genes was made, containing all the genes with ontology related to the immune system in the AmiGO2 database (51, 52), and all protein-coding genes interacting with known IEI genes, with a maximum distance of 2, according to the Human Reference Protein Interactome (RRID: SCR_01567) (53), the KEGG database (RRID: SCR_012773) (54), and the Human Genome Connectome (RRID: SCR_003490) (**Supplementary Table 1**). Variants in these genes were annotated and prioritized as described above, and then filtered and ranked based on criteria such as type and effect of the variant, gene function, signaling pathways and immune functions in which they take part, variant frequency in public databases, variant frequency in an in-house database, possible correlation with the phenotype of the patient, information on the role of the gene derived from laboratory models, and any published information related to the genes and their variants. For X-linked and autosomal recessive analyses, all variants and genotypes returned by the analyses were examined in detail; for the autosomal dominant analysis, the top 15 results were reviewed.

For the final stage, all variants in the exome were annotated and prioritized (**Supplementary Table 2**). All variants and genotypes were analyzed in detail for the X-linked and autosomal recessive modes of inheritance, and the top 25 variants and genes were analyzed when a monoallelic change was assumed.

All statistical tests were done with R (RRID: SCR_001905), version 4.3.1 (55).

## Results

From June 2015 to May 2024, 157 pediatric patients, with a suspicion of an IEI, were studied by NGS (**Supplementary Table 2**). The cohort had an average age of 6 years 10 months, and a median age of 5 years. As a requirement for sequencing, the paperwork accompanying the biological samples included a list of symptoms, a short clinical summary, laboratory test results, and a list of likely clinical diagnoses or genes of interest. The type, amount, and detail of this information varied widely from patient to patient. Examination of this information led to determine that 78.34% of the patients (123 of the 157) met the criteria for suspecting an IEI, according to the Jeffrey Modell Foundation (JMF) (**Supplementary Table 2**). The most common feature was infectious diseases. In 33 of the remaining patients the information was very limited and, lastly, another patient did not have at least two of the JMF criteria.

Initially, the TruSightOne (TSO) panel was used to obtain the sequencing library of nine patients. However, given the pace at which new disease genes were being reported, this panel was rapidly becoming outdated, and did not allow the re-analysis of the data for newly added IEI genes. For this reason, for the rest of the patients WES was done. In 45, of the 157 patients, a molecular diagnosis for an IEI was reached, representing a diagnostic yield of 28.66%. For the rest of the patients, all genes associated with pediatric disorders were analyzed, and a non-IEI disease was determined in five of them. In another patient, with an atypical mycobacterial infection, examination of the sequencing data, laboratory tests, clinical information, and follow-up, led to suggest that the patient might not have an IEI. Including these six patients in the calculation rises the diagnostic yield to 32.48%. Among the patients with a molecular diagnosis, a female had two diseases, DCLRE1C (Artemis) deficiency, and ichthyosis vulgaris. Also, in a patient with a non-IEI diagnosis, a digenic Alport syndrome was found.

**Figure 1:**
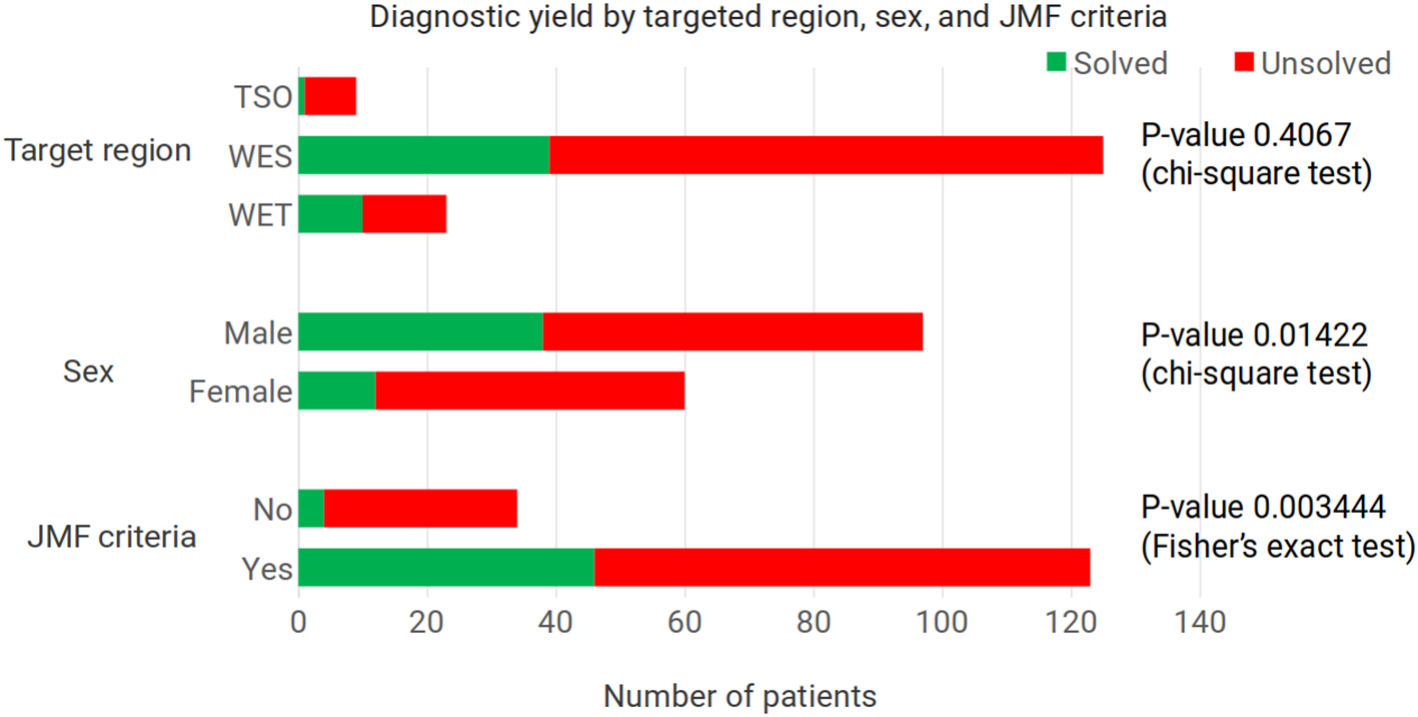
Diagnostic yield according to the enrichment kit used, the patients’ sex, and whether the patients met the criteria for an inborn error of immunity according to the Jeffrey Modell Foundation. For the target region, the P-value corresponds to the comparison of the two whole exome enrichment kits used. With all three enrichment kits, P-value = 0.2347, Fisher’s exact test. TSO – TruSightOne Targeted Regions panel v1.1, WES – Nextera Rapid Capture Exome Targeted Regions v1.2, WET – Twist ILMN Exome 2.0 or 2.5 Plus Panel, JMF - Jeffrey Modell Foundation.

While females comprised 38.22% of sequenced patients, they amounted to only 23.53% of the patients with a molecular diagnosis. In male patients the figures were 61.78% and 76.47%, respectively (**Figure 1**). This difference in diagnostic yield between sexes is statistically significant (P-value = 0.01422, chi-square test). Ten of the molecular diagnoses involved hemizygous genotypes in genes on the X chromosome in male patients; even when removing these patients, the proportion of molecular diagnoses in females was 29.27%, which is below the 38.22% proportion of females in the cohort. In males, the percentages, excluding X-linked diseases, were 70.73% and 61.78%, respectively.

With WES, the diagnostic yield was three times as high as with the TSO panel (33.78% versus 11.11%), although the number of patients sequenced with the latter panel was small (nine patients, versus 148 WES) (**Figure 1**). Separating the results for the two WES panels, with the Nextera Rapid Capture Exome Targeted Regions v1.2 the diagnostic yield was 32.00%, and with the Twist ILMN Exome 2.0 or 2.5 Plus Panel kits it was 43.48%. This difference was not statistically significant (P-value = 0.4067, chi-square test). We also checked whether the variants found using the Twist enrichment kit could have been found with the Illumina Nextera kit. Two of the variants found after using the Twist kit correspond to the homozygous loss of exons in genes where this defect is known to occur, *NCF1* and *RAB27A* (56–61); the complete absence of reads would have been apparent also with the Illumina Nextera enrichment kit. The remaining eight variants found in patients sequenced with the Twist kit were single nucleotide variants. We examined the coverage of these eight positions in 500 exomes sequenced with the Illumina Nextera kit, and we found good coverage for all of them. The position with the lowest depth in these 500 exomes corresponded to *KMT2D* chr12:49049834, with a mean depth of 51.19X (st dev 31.61); while the position with the highest depth in these 500 Exomes was found in *BTK* chrX:100611120, with a mean depth of 165.76 (st dev 86.56).

Results of the patients meeting the criteria of the JMF were compared against the set of patients where the information was insufficient to determine whether they met the criteria. In the group of patients meeting the criteria, 34.96% had a molecular diagnosis of an IEI. This contrasts with only 5.88% of IEI molecular diagnoses among the patients not meeting the JMF criteria (P-value = 0.0034444, Fisher’s exact test) (**Figure 1**).

**Figure 2:**
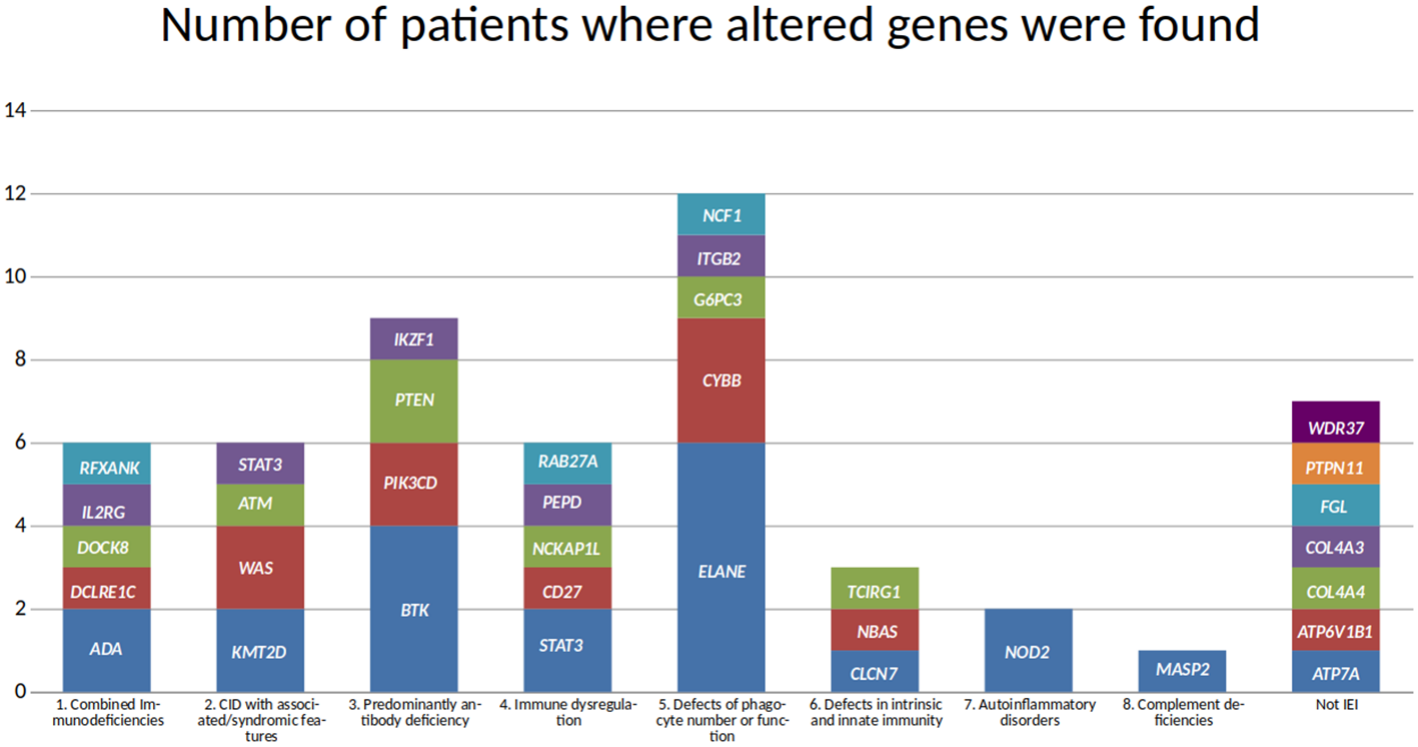
Genes found in the molecular diagnoses in the cohort. Genes are grouped according to the immunodeficiency disease group they are associated with, and the height of each block reflects the number of patients where the altered gene was found.

Following the IEI classification (6), 27 diseases were found in our cohort. Also, 27 different genes were associated with those diseases (**Figure 2**), although there is no biunivocal correspondence between the diseases and the genes (e.g., in *STAT3*, gain of function variants lead to STAT3 GOF, while loss of function variants are associated with AD-HIES STAT3 deficiency (Job syndrome); also, osteopetrosis appears as a single disease in the classification, but it may be caused by defects in any one of seven different genes).

The most common mode of inheritance of the molecularly diagnosed IEI was autosomal dominant (40.00%), closely followed by autosomal recessive (37.78%). X-linked diseases accounted for 22.22% of the molecular diagnoses.

The disease and gene most frequently seen in this cohort is neutropenia, caused by alterations in *ELANE*, in six patients. This was followed by variants in the *BTK* gene, associated with X-linked agammaglobulinemia, in four patients. *CYBB* and *STAT3* defects were each seen in three patients. However, only two of the patients with *CYBB* variants had X-linked chronic granulomatous disease associated with a missense variant. The third patient had a large deletion spanning approximately 1.8 Mbp, leading to the complete loss of many genes, including *CYBB* and *XK*, and a partial loss of *CFAP47* and *SYTL5*. Variants in *XK* have been associated with McLeod syndrome (OMIM 300842), while variants in *CFAP47* may be associated with Spermatogenic failure, X-linked 3 (OMIM 301059). Regarding the three patients with variants in *STAT3*, in two of them the phenotype corresponded to a gain of function, while in the third it was a loss of function. Even though several patients had variants in *ELANE*, *BTK*, *CYBB*, and *STAT3*, no two patients shared the same variant. Among the 27 IEI genes, only one variant was observed in more than one patient; a missense variant in *PIK3CD*.

Missense variants were the most frequent type of change (27 variants, including the *PIK3CD* variant observed twice), followed by eight stop gain variants. Copy number variants (CNV) were identified in three patients; one of them corresponding to the large hemizygous deletion affecting *CYBB* and several other genes, mentioned above. Another CNV involved a homozygous deletion in *NCF1*, and the third CNV comprised the homozygous loss of four exons of *RAB27A*.

Even though no two patients shared the same variant, apart from the *PIK3CD* missense variant, homozygous genotypes accounted for the great majority (70.59%) of the genotypes in autosomal recessive diseases (12 out of 17 patients).

Using the clinical and laboratory information attached to the samples, plus the clinically suspected disease, or the genes suspected as relevant, samples were assigned to one of the ten IEI groups recognized by the IUIS (6) (**Supplementary Table 2**). This is referred to as the “pre-NGS classification”. An extra group (labelled “undefined”) was added to accommodate samples with insufficient information to ascribe them to one of the IUIS groups. In some cases, the information associated with a sample pointed to, or was consistent with, more than one of the IUIS groups; these samples were also placed in the “undefined” group. This group was the largest, with 38.85% of the samples, followed by groups 2 (combined immunodeficiencies with associated or syndromic features; 14.01% of the samples), group 5 (congenital defects affecting phagocytes; 12.10% of the patients), and group 4 (immune dysregulation; 10.19%).

**Figure 3:**
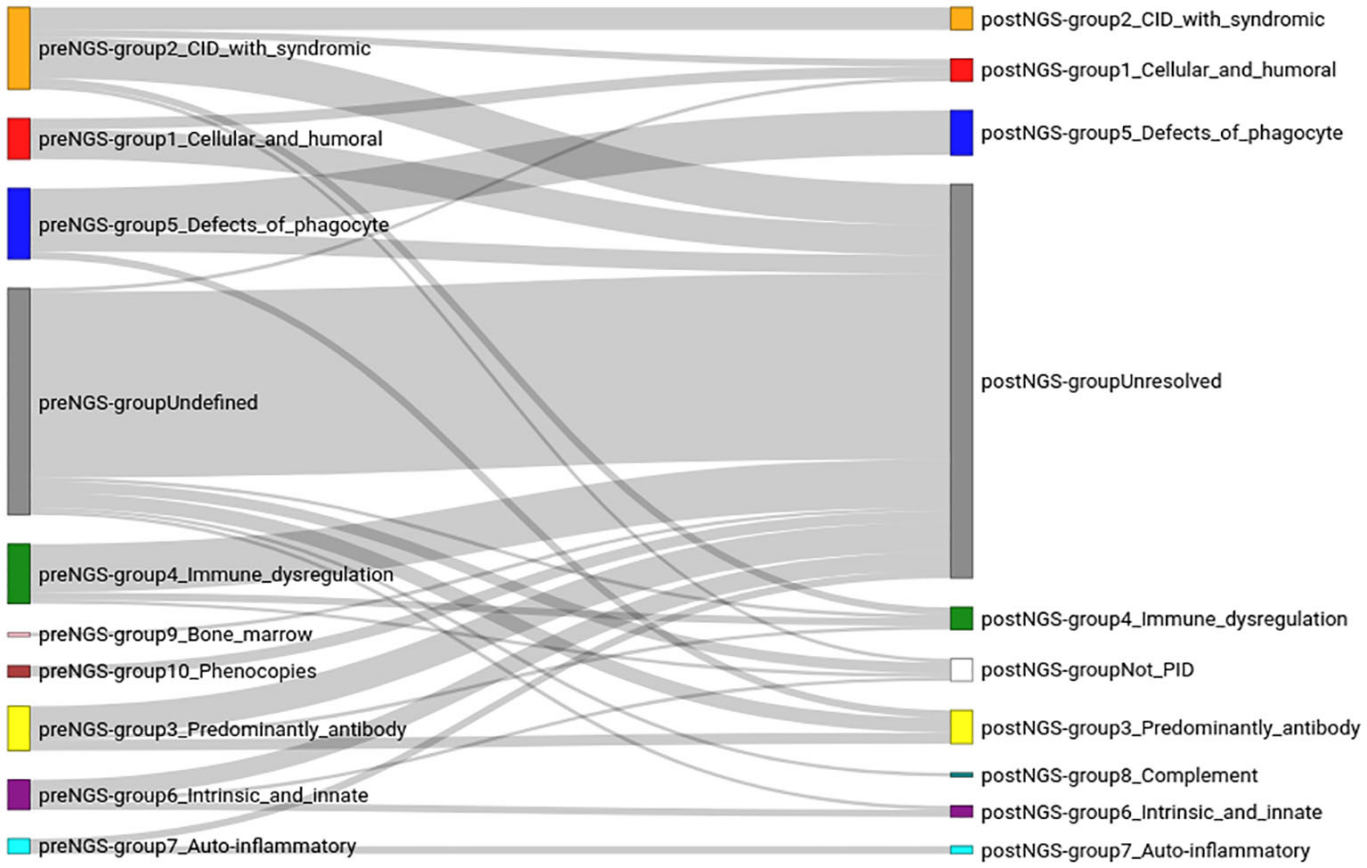
Classification of the patients in the cohort, before (preNGS) and after (postNGS) sequencing. The assignment of the immunodeficiency group before sequencing (left of the figure) was done based on the information provided by the clinicians (the clinical summary, results of some laboratory tests, and the clinician’s assessment of the likely disease of the patient). The right side of the figure corresponds to the immunodeficiency group after sequencing. The lines connecting the groups before and after sequencing reflect how the sequencing data changed the diagnosis of each patient.

With the results of the WES study, patients were again classified in the IUIS groups; this is the “post-NGS classification” (**Figure 3**; **Supplementary Tables 2, 3**). Two additional groups were considered: not-IEI, and “unresolved” cases. The largest group was formed by these unresolved cases (67.52%), followed by group 5 (congenital defects affecting phagocytes; 7.64% of the patients), and group 3 (antibody deficiencies, 5.73%). Some patients that were initially in the “undefined” group could be assigned to IUIS groups after NGS. However, most of the patients in the pre-NGS “undefined” group, ended in the post-NGS “unresolved” group. Patients in every group failed to have a molecular diagnosis after sequencing, and were assigned to the “unresolved” group. In some cases, such as in groups 1 to 4, most patients originally assigned to those groups failed to receive a molecular diagnosis.

The largest IUIS group, both pre-NGS and post-NGS, which also showed the highest concordance (63.16%) in group assignment, before and after sequencing, was group 5 (congenital defects affecting phagocytes), followed by group 7 (autoinflammatory disorders), although only four patients were initially thought to have a disease belonging to this group. Groups 2, 3 and 1 had a high pre-NGS number of patients (22, 12, and 11, respectively), and the concordance after sequencing was similar for all three of them (27.27%, 25.00%, and 27.27%, respectively). Group 4 also had an initial high number of patients, but the concordance after sequencing was lower (12.5%). No patients were initially thought to have a complement deficiency disease (group 8) but, after sequencing, one such patient was identified, who had initially been assigned to the “undefined” group.

**Table 1** summarizes the consequences of NGS in the diagnosis of IEI diseases. More than half of the patients who were initially assigned to an IEI group did not receive a molecular diagnosis. However, in slightly under a third of the patients the initial classification was confirmed by sequencing, and in ten percent of the patients the initial classification proved to be wrong, and a molecular diagnosis for a different IUIS group was determined. The table also shows that most (81.97%) of the patients who could not be assigned to an IEI group based on the information accompanying the sample, remained without diagnosis after being sequenced. Nevertheless, in 8% of the “undefined” patients sequencing proved useful in finding the correct disease.

**Table 1:** Contribution of next generation sequencing to the diagnosis of pediatric patients. IUIS classification of patients before and after sequencing.

Pre-NGS IUIS classification	Number of patients	Post-NGS IUIS classification	Number of patients	%
Defined IUIS group	96	Same IUIS group	30	31.25%
Defined IUIS group	96	Different IUIS group or non-IEI disease	10	10.42%
Defined IUIS group	96	Unresolved	56	58.33%
Undefined	61	Defined IUIS group or non-IEI	11	18.03%
Undefined	61	Unresolved	50	81.97%

IEI, inborn errors of immunity; IUIS, International Union of Immunological Societies; NGS, next generation sequencing.

As shown in **Table 1**, 40 patients had an assigned IEI group both pre-NGS and post-NGS; 50 patients were undefined/unresolved pre-NGS and post-NGS; 56 patients were assigned a defined IEI group pre-NGS but were “unresolved” post-NGS; and 11 patients had an undefined IEI pre-NGS, but could be assigned to an IUIS group post-NGS. These changes in patient classification after NGS are statistically significant (P-value = 7.639x10^-8^, McNemar test).

To search for new IEI candidate genes among the patients where no molecular diagnosis had been found, sixty patients were selected, based on the quality of their clinical and laboratory information. Variants in a curated list of 3,938 candidate genes were annotated, and then prioritized based on the HPO terms of the patients, and the information available for each gene. No strong new IEI candidate genes emerged. The reasons for this were varied. For example, some variants had a higher-than-expected frequency in public databases, or in our in-house database; for other variants, algorithms predicting the effect of the variant on the protein considered them as benign or likely benign, or the variants were in genes with functional information inconsistent with the phenotype of the patient. As a final step, variants were annotated, prioritized, and examined across the whole exome, and not only in the list of curated candidate genes. No strong new candidate genes were found after considering their frequency, the predicted consequence on the protein, and the known role of the gene.

## Discussion

We hereby present the results of the first large cohort of pediatric patients, in Mexico, with a suspected IEI, to be studied by next generation sequencing. In all 157 patients, except nine, the whole exome was sequenced. The diagnostic yield, just under 30%, was similar to those reported for other cohorts with heterogeneous IEI (9, 13, 19). The cohort comprised 1.6 times as many male as female patients. However, the proportion among those where a molecular diagnosis was determined was 3.25 diagnosed males for each diagnosed female. The source of this wide gap is unclear, since it cannot be accounted for by X-linked diagnoses. Even excluding all patients with diseases having this mode of inheritance, which were all hemizygous males, there were 2.42 diagnosed males per diagnosed female.

Even though the diagnostic yield in IEI is relatively low, WES is clearly valuable. Almost one in five of the patients that lacked a defined immune disease when they were submitted for sequencing, received a molecular diagnosis, while one in ten of the patients with an IEI of a specific group before sequencing, received a molecular diagnosis of a disease belonging to a different group after sequencing. In addition to this, slightly under a third of the patients that were submitted with a suggested immune disease, had a disease belonging to the same group, according to the IUIS classification, once the molecular diagnosis was determined.

From the clinical information accompanying the samples that were received for sequencing, slightly more than two thirds of the patients qualified as having an IEI according to the criteria of the JMF. The molecular diagnosis was found in approximately one in three of these patients, compared to only one in twenty in the group of patients where the JMF criteria were not met. The reason for this difference is unclear. It does not necessarily mean that the latter group of patients lacked an IEI, given that for almost all of them, failure to meet the criteria for an IEI, according to the JMF, was due to a lack of available information, rather than the phenotype being inconsistent with an IEI. Had this last possibility been the case, it would have been expected to find more non-IEI diagnoses when analyzing variants across the whole exome. This was not the case and, in fact, more non-immune diseases were found among the patients meeting the JMF criteria, than among those that did not.

Twenty-seven different IEI were found, and the same number of different genes. The most common disease was neutropenia due to monoallelic variants in *ELANE*, in six patients, followed by X-linked agammaglobulinemia in four patients, and X-linked chronic granulomatous disease in three. Even though some patients had the same disease, only two patients in the cohort shared the same variant, a missense change in *PIK3CD*. This underscores the rarity of the variants associated with these diseases in our population. However, in contrast to this, homozygous genotypes were observed in most of the patients with an autosomal recessive disease; most of these children came from small, and relatively isolated, rural communities.

Missense variants accounted for the vast majority of the genetic defects, while only three CNVs were found. It is possible that these latter variants are underrepresented, given the difficulty of reliably identifying them with exome sequencing (62). It was possible to find the three CNVs because they were homozygous deletions in two of the patients (involving *NCF1* and *RAB27A*), while the third, affecting *CYBB*, was hemizygous. Deletions affecting *CYBB* and *NCF1* are well known, even if the breakpoints differ between affected patients (56, 58, 63–65).

In our patients with neutropenia associated with variants in *ELANE*, all variants, except one, occurred in exons four and five. This is consistent with the findings reported in the literature (66). It is known that there is some genotype-phenotype correlation, with some variants found in severe congenital neutropenia, and others in cyclic neutropenia, although a few variants have been found in both diseases (67). Among the six variants we identified, Gly214Arg has been observed repeatedly in patients with neutropenia. This variant is in a mutation hotspot and is associated with poor prognosis (66–68). Patients with this variant are at increased risk of presenting myelodysplastic syndrome and acute lymphoblastic leukemia. The Gly214 residue is evolutionary conserved, and the change affects the conformation and stability of the polypeptide chain (69). All possible changes in this position (c.640G>A, c.640G>C, c.640G>T) have been reported in patients with neutropenia (70). The Gly214Arg change has been the subject of functional studies, and it is known that the misfolded protein triggers the unfolded protein response, and results in apoptosis (71–73). The possibility of using inhibitors of this misfolded protein has been studied *in vitro* (74). In our cohort, the patient with this variant received the molecular diagnosis when she was 4 months old. She has seen again a few months later, but she did not come back to the hospital so it is unknown how her disease developed. She would be almost seven years old today. The rest of the *ELANE* variants in our cohort have not been the object of detailed studies. Two of them, Leu227SerfsTer13 and Tyr228Ter (70), are towards the C-terminus of the protein, and it is predicted that they would escape the nonsense-mediated decay process (75, 76). We found two *ELANE* inframe deletions, one in exon two leading to the predicted loss of six aminoacid residues, and the other in exon four, predicted to result in the loss of two residues. Another variant was a missense change in exon 5 (Gly210Trp) (70); this variant, as well as the former four mentioned above, has not been studied in detail. In our group of neutropenia patients associated with *ELANE* alterations, the most frequent clinical manifestation was infections, and all patients had at least one elevated Ig class. In all of them, the first symptoms appeared during the first months of life and they comprised recurrent pneumonia, otitis media, and neonatal sepsis. The most common infections were associated with *Pseudomonas aeruginosa* and *Escherichia coli*. No differences of note were observed between the six patients. From the very beginning, based on the clinical examination and laboratory tests, a clinical diagnosis of neutropenia was suggested, and this diagnosis was confirmed by sequencing.

Four patients in our cohort had *BTK* variants. Three of them were stop codon gains, and the fourth was a missense variant. One of the stop codons was in the TH domain of the protein and the affected patient showed a more acute clinical presentation of the disease than the other three cases. This patient had very severe infections and depletion of all lymphocyte subpopulations. Initially, he also had hemophagocytic lymphohistiocytosis. Due to the severity of the clinical signs, chronic granulomatous disease and severe combined immunodeficiency were suspected. The rest of patients with *BTK* alterations had variants in the kinase domain of the protein. All three of them had typical symptoms of the disease, including agammaglobulinemia, and severe infections as the initial symptoms.

The three patients with *CYBB* variants, and a diagnosis of chronic granulomatous disease, shared the same features, with a severe presentation, BCGitis, recurrent pneumonia, sinopulmonary infections, and adenitis. Two of the patients had stop gain variants, in codons 157 and 226. The third patient had a large deletion, leading to the complete absence of *CYBB* sequences, in addition to the loss of at least 30 other genes, including 11 protein coding genes. One breakpoint was located between exons 35 and 36 of *CFAP47*, and the other between exons 15 and 16 of *SYTL5*. Only a few of the genes in this region have been associated with a disease. Deletions in this region, leading to the complete loss of *CYBB* and other genes, are known (77). The extent of the deletion may differ from one patient to another and the phenotype of the patient depends on which genes are lost. This may cause patients to have more than one disease (63, 64, 78). However, in our patient, even though many genes besides *CYBB* were missing, his disease showed no differences when compared to our other two patients, who had single nucleotide variants causing a stop codon gain. No other phenotypes or diseases, stemming from the loss of genes besides *CYBB*, were observed. Nevertheless, it is still possible that additional phenotypes may appear at a later age. For example, in this patient the first exons of *CFAP47* are missing, and alterations in this gene have been associated with azoospermia (79). Also, alterations, most frequently deletions, in the *XK* gene have been associated with McLeod syndrome, including late onset myopathy and neuropathy (80–83). In some patients with McLeod syndrome due to large deletions, *VPS13B* and *DMD* may be lost. However, these two genes were present in our patient.

Looking at the frequency of the different types of IEI in this cohort, both before and after sequencing, points to a predominance of patients with diseases belonging to group 5 (congenital defects of phagocyte number or function), group 3 (predominantly antibody deficiencies), and group 2 (CIDs with associated or syndromic features). However, this distribution does not necessarily reflect the frequency of the different types of IEI in our country, or even in our hospital. In our institution, there is some bias in the patients submitted for sequencing. Clinicians tend to send patients where they struggle to identify the immune disease; they also send patients requiring a precise molecular diagnosis to proceed with the correct treatment, or very ill inpatients where a precise diagnosis is required as quickly as possible to decide on the best course of action. In the cohort we report, one in ten children had an IEI of a different group than initially thought, while close to 40% had an “undefined” immune disease before sequencing. These figures point to the difficulty of correctly identifying these diseases where symptoms are non-specific and overlap between diseases. In addition to this, in this hospital, there is a very limited number of laboratory tests and studies available to perform on these patients. These factors are an impediment to a precise and correct diagnosis. In this regard, NGS has become an indispensable tool. On the other hand, the proposition that patients, in our hospital, for which no sequencing is sought, have a correct diagnosis, is something that needs to be tested, by sequencing them, and by assessing the concordance between the diagnoses suggested by the clinicians relying on the limited set of laboratory tests available at the institution, and the molecular diagnoses after sequencing (pre-NGS *versus* post-NGS comparison).

Among the patients where no molecular diagnoses were found, new IEI candidate genes were searched. However, no candidates were identified, first by analyzing variants and genes in a curated set of immune related genes, and then by studying the variants across the whole exome. This negative result is not entirely unexpected. In WES, many factors may be responsible for negative results, without necessarily implying that new genes are involved. CNVs, segmental duplications, structural variants, intronic alterations and intergenic variants, are missed when sequencing exonic coding regions in patients with immune diseases (62, 65, 84–86). In addition to this, GC-rich regions, often found in gene regulatory regions, are difficult to sequence, and sequence drop out (regions with zero coverage) may occur. CNVs have been reported in a number of IEI (62). In our cohort, seven patients suspected of having neutropenia remain unresolved. This disease may sometimes be the result of copy number variants, due to deletions, in *ELANE* (66, 70, 87), so this possibility should be explored, as well as the possibility of variants in non-coding regions, leading to altered transcripts and proteins (88). It has also been found than some patients with neutropenia have somatic variants in *ELANE*, as opposed to germline alterations (87). Our cohort also includes a patient with Wiskott-Aldrich where no variants were found after carefully scanning the reads covering all coding exons. In this patient, sequencing of the gene’s regulatory region, analysis of the gene’s transcript, by qRT-PCR, or protein studies by Western blot, may allow to determine if there is a variant affecting not the protein sequence, but the expression of the gene.

Even though the literature mentions that the *PIK3CD* pathogenic variant E102K may be missed due to a duplicated sequence (84), we had no difficulty finding this variant in our cohort. It was, in fact, the only variant that we found in more than one patient.

Our cohort also includes eight patients where hypereosinophilic syndrome was suspected. One of them was found to have a *STAT3* gain of function variant. In the remaining seven, no alterations could be identified. It is known that hypereosinophilic syndrome is associated with structural variants, including chromosome translocations, leading to gene fusions, with *FIP1L1* and *PDGFRA* being the genes most frequently implicated (89, 90). In addition to this, somatic variants in *JAK1* and *STAT5B* have been reported. In these patients, the molecular alterations could have been missed because exome sequencing is unable to find structural variants, unless the breakpoints occur within the coding exons, and they could also have been missed if the disease was caused by somatic alterations.

Among the patients with no molecular diagnosis in our cohort, there are nine where CVID is suspected. The diagnostic yield is low in patients with these diseases, and it has been suggested that it might be necessary to consider non-monogenic genetic architectures, such as digenic, oligogenic, the participation of several common variants affecting specific pathways, or even genetic models corresponding to complex diseases where an external triggering factor plays a role (3, 85, 86). Additionally, epigenetic modifications may also be implicated in IEI, although this area appears to have received scant attention (91). These studies, and the fact that with NGS the diagnostic yield in IEI is lower than for other monogenic diseases, suggest that there is indeed a difference in the assumed genetic architecture of immune diseases (92, 93).

In our hospital, WES is the technique of choice to determine the correct disease in patients with IEI, and to identify the precise variant underlying the disease. We have shown here that this approach leads to the molecular diagnosis of patients where the clinical history, the clinical examination, and laboratory tests are insufficient to suggest a specific IEI disease. We have also shown that patients where a clinical diagnosis has been suggested may, in fact, have a different disease to the one initially considered. Even though WES has proven its usefulness, it has limitations and pathogenic variants may be missed. Relying on WGS, instead of exome sequencing, would likely lead to a higher diagnostic yield, mainly through the detection of CNVs and structural variants. While variants in non-coding regions can also be found by WGS, the difficulty in interpreting these variants means they do not in fact contribute very much to increasing the diagnostic yield. This is true even though bioinformatic tools, such as REMM (RRID: SCR_023095) (94) and CADD (RRID: SCR_018393) (95), have been developed to aid in the interpretation of non-coding variants. For this type of variants, transcriptome sequencing may be preferrable (96). Transcriptome sequencing has the added advantage of providing functional information, on top of the sequencing data itself. Up to now, we have limited ourselves to WES mainly due to cost constraints. However, in order to increase the diagnostic yield in patients with IEI, we are expecting to implement a stratified approach, where WES would be the first technique to use, followed by WGS in patients where no molecular diagnosis is found. This would make it possible to increase the diagnostic yield by 5-10% by enabling the detection of CNVs and structural variants (62). WGS, if performed with long read sequencing, would also enable phasing, and solve segmental duplications that might be difficult to resolve correctly with short read sequencing. In patients where no molecular cause is found after WGS, we would use transcriptome sequencing to look for variants in non-coding regions affecting splicing. Combining transcriptome and WGS data should also aid in the interpretation of non-coding variants affecting gene expression (97).

We conclude that, even though NGS has proven its usefulness in the diagnosis of IEI, more needs to be done if we are to increase the currently low diagnostic yield, and this means broadening the technological and bioinformatics tools in use, and making more progress in understanding the genetic architecture of these diseases.

## Data Availability

The original contributions presented in the study are publicly available. This data can be found in ClinVar submission SUB15651872 (https://www.ncbi.nlm.nih.gov/clinvar/?term=SUB15651872), accession numbers SCV006554572 to SCV006554623.
